# Can Primary School Mathematics Performance Be Predicted by Longitudinal Changes in Physical Fitness and Activity Indicators?

**DOI:** 10.3389/fpsyg.2022.796838

**Published:** 2022-02-08

**Authors:** Vedrana Sember, Gregor Jurak, Gregor Starc, Shawnda A. Morrison

**Affiliations:** Faculty of Sport, University of Ljubljana, Ljubljana, Slovenia

**Keywords:** accelerometers, physical fitness surveillance, paediatric exercise, health-related risk factors, population health, arithmetic, children

## Abstract

**Objective:**

To determine to what extent physical fitness indicators and/or moderate to vigorous physical activity (MVPA) may account for final mathematics academic performance (AP_math_) awarded at the end of primary school.

**Methods:**

School-aged youth were sampled in a repeated-measures, longitudinal design in Grade 6 (∼11 years), and again in Grade 9 (∼14 years). The youth (*N* = 231, 111 girls) completed a fitness test battery consisting of: flamingo balance test, standing long jump, backward obstacle course, plate tapping, sit ups, sit and reach, handgrip, and 20-m shuttle run. AP_math_ scores were obtained for all children at the end of Grade 5, end of Grade 8, and end of Grade 9 (their final year of primary school). In a sub-sample of Grade 6 youth (*N* = 50, 29 girls), MVPA was measured objectively via SenseWear Pro Armbands (MVPA_OB_) for seven consecutive days, with measurements repeated in Grade 9.

**Results:**

Math scores decreased from Grade 6 to 9 for both boys and girls (95%CI: −0.89 to −0.53, *p* < 0.001). MVPA_OB_ was reduced by ∼45.7 min (−33%) from Grade 6 to 9 (*p* < 0.01). Significant main and interaction effects are noted for each fitness indicator (*p* < 0.05). A backward stepwise multiple regression analysis determined significant shared variance in final AP_math_ grade to the change scores from Grade 6 to Grade 9 in: ΔAP_math_, Δbackward obstacle course, Δsit and reach, and Δsit-ups [*R*^2^ = 0.494, *F*(4,180) = 43.67, *p* < 0.0001]. A second regression was performed only for the youth who completed MVPA_OB_ measurements. In this sub-sample, MVPA_OB_ did not significantly contribute to the model.

**Conclusion:**

Longitudinal changes in youth fitness and their delta change in AP_math_ score accounted for 49.4% of the variance in the final math grade awarded at the end of Grade 9. Aerobic power, upper body strength, and muscular endurance share more common variance to final math grade in boys, whereas whole-body coordination was the more relevant index in girls; this finding suggests that future research exploring the relationship of AP and PF should not be limited to cardiorespiratory fitness, instead encompassing muscular and neuro-muscular components of PF.

## Introduction

Physical activity (PA) is a dynamic state of being (Reid et al., 2019) which can be defined as any bodily movement produced by skeletal muscles resulting in energy expenditure (Caspersen et al., 1985) greater than that which exceeds hibernation (Fletcher et al., 1996). Participating in regular PA is indispensable to maintaining a healthy lifestyle, in part because of the concomitant positive impacts on skeletal (Gunter et al., 2012), metabolic (Janssen and LeBlanc, 2010), cardiovascular (Fernhall and Agiovlasitis, 2008), and psychosocial functioning of the human body (Biddle and Asare, 2011). Attaining low levels of PA and the attendant poor aerobic fitness is associated with declines in academic performance (AP) (Chaddock et al., 2011), the deterioration of brain structures, cognitive abilities, and general brain function (Sibley and Etnier, 2003; Castelli et al., 2007; Chaddock et al., 2010; Donnelly et al., 2016; García-Hermoso et al., 2021). Increasing PA has therefore, long been suggested to exert a positive impact on AP, especially since learning complex movements stimulates the frontal cortex of the brain, which is also active in the learning and problem-solving process (Daly-Smith et al., 2018; Singh et al., 2019; Duffey et al., 2021).

There is a growing body of work which have found significant relationships between PA and AP (Chaddock-Heyman et al., 2013; Daly-Smith et al., 2018; Sember et al., 2020b). However, the positive effects of PA on AP may accrue most readily only when there is an adequate amount of vigorous PA included (Tomporowski et al., 2011; Phillips et al., 2015). Evidence regarding the association between PA and any broader aspect(s) of AP have (thus far) remained ambiguous, with some researchers also finding negative, or null effects in this relationship (Coe et al., 2006; Beck et al., 2016; Riley et al., 2016). These inconsistent findings may be due to a difficulty in precisely assessing both the overall amount, and intensity of, PA, which children and adolescents regularly undertake (Monyeki et al., 2018; Sember et al., 2020a).

The level of physical fitness (PF) for a given individual is related to a great many factors, including the outcome of their habitual PA habits, genetics, socio-economic status, and environment, among others. Physical fitness can be highly informative when examining the health effects of PA and AP (Sardinha et al., 2014), including recent evidence that cardiorespiratory fitness is associated with larger brains during a critical phase in children, when the brain is growing (Cadenas-Sanchez et al., 2020). For example, it has been shown that after-school PA improves children’s cardiovascular endurance, which then mediates enhancements in AP (Fredericks et al., 2006). A meta-analysis (including articles from 2005 to 2015), which investigated the relationship between PF and AP asserted that cardiorespiratory fitness, speed-agility, motor coordination, and perceptual-motor skills are each highly associated fitness indicators to AP (Ruiz-Ariza et al., 2017). Moreover, several studies have identified positive relationships between cardiorespiratory fitness, body mass and AP (Eveland-sayers et al., 2009; Van Dusen et al., 2011; Rauner et al., 2013; Torrijos-Niño et al., 2014) or overall PF (Starc et al., 2017). Findings on the relationship of AP to strength and flexibility remain unclear (Chaddock-Heyman et al., 2014; Tannehill et al., 2015; Ruiz-Ariza et al., 2017; Esteban-Cornejo et al., 2019).

Although there is an abundance of scientific papers dealing with the relationship between PA and cognitive function, researchers often use indirect assessments of PA in their work. Indeed, although researchers and public health policies continue to cite PA as a primary movement/health-based indicator, it is also a highly individual one, where any two individuals may need varying doses (i.e., frequency, duration, intensity, and type of activity) to achieve the same physiological and/or health related benefits as one another (Reid et al., 2019). Notably, objective PA assessment is often not feasible for large studies involving hundreds or even thousands of participants, due to its high cost and complications arising from extracting PA data from the measuring devices (Dowd et al., 2018). Although several studies have found a significant relationship between aerobic function and AP, most have not considered longitudinal changes in PA and PF across their sample.

Thus, the purpose of this study was four-fold: (1) to determine longitudinal changes in PF, MVPA (measured objectively) and AP_math_ in a cohort of Slovenian youth across a 3-year timespan, (2) to determine whether individual PF indicators were correlated to MVPA or AP_math_ scores within a given Grade, (3) to determine whether changes in MVPA or PF indicators related to changes observed in AP_math_, and (4) to what extent could these changes over time account for final AP_math_ score attained at the end of primary school. It was hypothesized that youth who were able to maintain higher PA and PF would maintain or achieve higher math scores at the end of their schooling period compared to those who were less active or fit.

## Materials and Methods

### Research Design and Study Sample

We obtained the data for this longitudinal, cross-sectional study within *The Analysis of Children’s Development in Slovenia* (ACDSi), an ongoing study used to monitor the physical and motor development of children and youth in Slovenia every 10 years for the last five decades (Jurak et al., 2013; Starc et al., 2015). The ACDSi study was approved by the Slovenian National Medical Ethics Committee (ID:138/05/13), following the Declaration of Helsinki. We obtained written, informed, parental consent and child assent before testing for each child involved in the study. Children and youth participated voluntarily and anonymously and had the option of freely withdrawing from the measurements at any time. The ACDSi study uses a sentinel approach to provide a nationally representative sample of children from 11 different locations, stratified according to their environment (e.g., village, town, industrial town, and city). The primary sampling unit is the school, and the secondary one defined as the schoolchildren’s class. At each of the 11 primary school locations, research teams were divided into three test stations: anthropometry, motor and aerobic fitness and psychology. The research team from the Faculty of Sports, University of Ljubljana collected data for this study in autumn 2013, then ∼3 years later during summer of 2016, and again in autumn 2016. In the 2013 generation of the ACDSi study, the Grade 6 sample included 231 youth (120 boys and 111 girls), aged 11 years old (born on October 01, 2002, ±6 months). The average age of all youth included in the Grade 6 sample was 11.29 ± 0.30 years (boys 11.3 ± 0.32; girls 11.29 ± 0.27), and the average age of the youth wearing SWA was 11.23 ± 0.3 years (boys 11.24 ± 0.36; girls 11.22 ± 0.25). All participants were measured again, exactly 3 years later, in Grade 9.

### Experimental Measures

#### Physical Activity

We assessed MVPA objectively on a sub-sample of children (*N* = 50, boys = 21 girls = 29) wearing a multi-sensor device (SWA, Bodymedia SenseWear Pro Armband; BodyMedia Inc., Pittsburgh, PA, United States, MVPA_OB_). Device specifications, data collection techniques, and standard methodology is outlined in detail elsewhere (Sember et al., 2020a). The SWA device is based on the recognition of energy expenditure patterns resulting in an estimation of PA and is considered to be a reliable measuring device in children and youth (Calabró et al., 2009; Soric et al., 2013; Stålesen et al., 2016). Participants wore the SWA on their triceps (i.e., right side of upper arm) for 1 week (Cain et al., 2013), 24 h per day, except when they were showering, bathing, or performing water-based, or sporting activities (e.g., swimming and/or competitions in other sports).

#### Physical Fitness

We assessed PF via the following indicators: 20-m shuttle run (peak aerobic power, $\dot{\text{V}}\,{\text{O}}_{2\,p\,e\,a\,k}$), polygon backward obstacle course (coordination), handgrip (muscular strength), standing long jump (power), flamingo test (balance), sit-ups (repetitive strength) plate tapping (reaction time) and sit and reach (hip joint flexibility). Details on test protocols are available elsewhere (Jurak et al., 2013; Starc et al., 2015). $\dot{\text{V}}\,{\text{O}}_{2\,p\,e\,a\,k}$ was estimated using Mahar’s prediction equation (Mahar et al., 2011), estimated from the 20-m multistage shuttle run performance test, which was conducted following Leger’s original test protocol (Leger and Lambert, 1982). Jamar’s dynamometer (TEC, Clifton, NJ, United States) was used to measure handgrip strength on the child’s dominant hand.

#### Mathematics Performance

The indicator taken to best reflect AP in Slovenian schoolchildren and youth was their math grade, awarded by their teachers at the end of the previous academic year. Math grades were recorded for all youth representing the start of Grade 6 (i.e., final score awarded at end of Grade 5), the start of Grade 9 (final score awarded at end of Grade 8), and their final mathematics mark, awarded at the end of Grade 9. The math grade represents a modest indicator of overall AP in the literature, but it is especially appropriate for the Slovenian setting, with an *r* = 0.50 (Flere et al., 2009) and higher levels of reliability (0.89–0.94) (Carlson et al., 2008) than other subjects or language scores (e.g., Slovenian or English), for example. In Slovenia, math grades are awarded following next-order methodology: 1 (inadequate), 2 (sufficient), 3 (good), 4 (very good) and 5 (excellent).

### Data Processing

Objective physical activity (MVPA_OB_) assessment of moderate-to-vigorous physical activity (MVPA) were analyzed using the Bodymedia SenseWear Professional 8.1 Software package and scored/adjusted manually where necessary. Microsoft Excel 2007 was used for removing artifacts, counts >16,000, constant values ≥0, and sequences of zeroes, where a sequence was defined as 20 or more zeroes. MVPA_OB_ data was processed with BodyMedia SenseWear Armband software 3.0 (standardized for accelerometer SenseWear Armband), following the 70/80 rule (Catellier et al., 2005) and non-wear time within day (Troiano et al., 2008). We only included the data of participants who wore the SWA device at least 5 days in a row, including both weekend days, and whose wear time exceeded 90%, following methods described elsewhere (Cain et al., 2013). The SWA data was collected in 1-min epoch intervals. The main rationale for using this conservative approach was to reduce the error of under- and over-estimation of PA due to missing wear-time, often an issue in current PA child health studies. Finally, difference scores of fitness and activity indices were calculated between Grade 6 and Grade 9 before statistical analyses were conducted.

### Statistical Analyses

We conducted all statistical analyses using SPSS version 27.0 (IBM Inc., Armonk, NY, United States). Data are presented as means and standard deviation, with 95% confidence intervals (CI), *F*-ratios and effect size where appropriate. We determined normality of distribution using the Kolmogorov–Smirnov test and Shapiro–Wilk test. Data were checked for multicollinearity and normality. Longitudinal changes in physical fitness and activity levels from Grade 6 to Grade 9 were compared using a two-way repeated-measures analysis of variance (RM ANOVA), with one within-subjects factor (time, two levels: Grade 6 and Grade 9) and one between-subjects factor (sex, two levels: male, and female), as well as the non-parametric alternative conducted for AP_math_ (Friedman test), set at the *p* < 0.05 level of significance. Two-tailed, bivariate correlations were run to query whether fitness indicators were related to MVPA_OB_ using Pearson’s test, calculated for each grade separately. Correlation coefficients between AP_math_, MVPA_OB_, and fitness variables were conducted using Spearman’s rho (ρ). A backward, stepwise multiple regression was conducted on the entire sample of children to determine to what extent changes in individual fitness indicators shared common variance to final Grade 9 AP_math_ scores. The procedure was then conducted separately for the *N* = 50 MVPA_OB_ sub-sample with ΔMVPA_OB_ between years as an input variable. Statistical criteria for a given index’s inclusion in the model was set at the *p* < 0.05 level, and omission ‘out’ occurred when an index posted *p* > 0.10 during the backward step analysis; missing cases were dropped in a listwise fashion.

## Results

### Longitudinal Changes in Physical Activity, Fitness, and Mathematics Academic Performance

Objectively assessed physical activity (MVPA_OB_) decreased ∼33% (i.e., −45.7 min) from Grade 6 to Grade 9 [main effect: *F*(1,49) = 6.225, *p* = 0.016, Table 1], including a significant interaction effect by sex (CI: 10.1–72.2 min, *p* = 0.010).

**TABLE 1:** Descriptive statistics (means ± sd) of physical activity, fitness, and academic performance (math grade).

		MVPA_OB_	Shuttle run	Polygon	Standing long	Handgrip	Flamingo	Sit-ups	Plate tapping	Sit and	AP_Math_
		(minutes/day)	$\dot{\text{V}}\,{\text{O}}_{2\,p\,e\,a\,k}$	backward (s)	jump (cm)	(Nm)	(# falls/60 s)	(#/60 s)	(# of taps/20 s)	reach (cm)	(score)
Grade 6	Combined (*n* = 231)	138.9 + 73.2	46.0 + 26.5	16.3 ± 5.1	157.4 + 21.9	20.9 + 4.2	15.9 + 7.5	37 ± 9	35.5 ± 10.2	19.3 ± 6.9	4.2 + 0.8^1^
	Boys (*n* = 120)	149.4 + 72.5	45.8 + 26.3	15.9 ± 4.6	160.4 + 21.3	21.1 + 4.2	16.3 + 7.4	39 ± 9	36.3 ± 10.7	17.2 ± 6.2	4.1 + 0.09^1^
	Girls (*n* = 111)	128.2 ± 72.9^†^	46.2 ± 26.9	16.7 ± 5.1	154.2 + 22.3	20.7 + 4.3	15.4 + 7.7	35 ± 8	34.6 ± 9.5	21.5 ± 7.1†	4.2 + 0.8^1^
Grade 9	Combined (*n* = 231)	93.2 + 62.0*	53.25 + 25.1*	13.6 ± 4.3*	176.7 + 32.3*	28.9 + 4.2*	16.5 + 13.5	41 ± 10*	42.0 ± 5.7*	28.4 ± 9.5*	3.5+1.0*^2^
	Boys (*n* = 120)	123.6 + 65.6*	63.8 + 24.6*	13.1 ± 4.1*	187.7 + 31.8*	30.8 + 7.6*	17.4 + 18.6	44 ± 11*	41.8 ± 6.5*	24.9 ± 8.3*	3.4 + 1.0*^2^
	Girls (*n* = 111)	69.8 + 47.7*^†^	41.8 + 20.2*^†^	14.0 ± 4.4*	164.8 + 28.6*^†^	27.0 + 5.2*^†^	12.8 + 10.6*^†^	38 ± 9*	42.3 ± 4.5*	32.3 ± 9.2*^†^	3.5 + 1.0*^†2^
End of Grade 9	Combined (*n* = 231)										3.5 + 1.0^3^
	Boys (*n* = 120)										3.4 + 1.1^3^
	Girls (*n* = 111)										3.6 + 1.0 ^3^

Aerobic power calculated from 20 m shuttle run ($\dot{\text{V}}\,{\text{O}}_{2\,p\,e\,a\,k}$) following the Leger protocol (Leger and Lambert, 1982), expressed in mL⋅kg^–1^⋅min^–1^. MVPA_OB_, physical activity measured directly using SenseWear Armbands in an N = 50 sub-sample (N = 21 boys and 29 girls); AP_math_, academic performance assessed via math grade, ^1^corresponding to the final grade awarded at the end of Grade 5, ^2^at the end of Grade 8 and ^3^at the end of Grade 9. *Significantly different from Grade 6 (p < 0.05); ^†^Significantly different from boys within that grade level (p < 0.05).

There were significant main effects for time (*p* < 0.05, Table 1) in all fitness variables, for both girls and boys. By Grade 9, there were also numerous significant differences between girls and boys within that grade; the 95% confidence intervals showed that boys produced more aerobic power (measured via shuttle run, CI: 5.9–15.7 mL⋅kg^–1^⋅min^–1^, *p* < 0.0001), achieved better results in the standing long jump (CI: 8.2–20.7 cm, *p* < 0.0001), produced more force in handgrip (CI: 0.8–3.3 Nm, *p* = 0.001), and balanced longer during flamingo testing (CI: 0.1–5.3 s, *p* = 0.042), whereas girls demonstrated greater hip and lower back flexibility with higher sit and reach scores (CI: 5.0–9.6 cm, *p* < 0.0001).

In terms of the longitudinal results of academic performance, assessed by mathematics grade (AP_math_), this measure decreased from Grade 6 to Grade 9 for all youth (CI: −0.89 to −0.53, *p* < 0.0001) equivalent to ∼0.7 of a grade (i.e., −17%). The magnitude drop did not differ between boys and girls (CI: −0.32 to 0.04, *p* = 0.120).

### Correlations

#### Physical Activity and Fitness

For Grade 6 youth, standing long jump (*r* = 0.193, *p* = 0.44) backward obstacle course (*r* = 0.208, *p* = 0.030), flamingo (*r* = −0.232, *p* = 0.015) and sit-ups (*r* = 0.213, *p* = 0.025) fitness scores were moderately correlated to MVPA_OB_ (Table 2). When fitness indicators were separated by sex, fitness variables correlating to MVPA_OB_ differed, with only flamingo test demonstrating a moderate correlation (*r* = 0.375, *p* = 0.005) in boys, and no individual variable reaching significance for girls. By Grade 9, the strength of these linear associations had increased, such that MVPA_OB_ was positively correlated to standing long jump (*r* = 0.305, *p* = 0.002), sit-ups (*r* = 0.288, *p* = 0.004), handgrip (*r* = 0.251, *p* = 0.011), shuttle run (*r* = 0.513, *p* < 0.0001), and backward obstacle course (*r* = 0.283, *p* = 0.004).

**TABLE 2A:** Correlation coefficients for dependent measures determined for boys and girls in Grade 6.

Grade 6		MVPA_OB_	Shuttle run $\dot{\text{V}}\,{\text{O}}_{2\,p\,e\,a\,k}$	Polygon backward	Standing long jump	Handgrip	Flamingo	Sit-ups	Plate tapping	Sit and Reach	AP_Math_
MVPA_OB_ (*r*)	All Boys Girls										
Shuttle run $\dot{\text{V}}\,{\text{O}}_{2\,p\,e\,a\,k}$	All Boys Girls	0.104 0.033 0.188									
Polygon backward	All Boys Girls	−0.208* −0.257 −0.172	−0.098 −0.163 −0.064								
Standing long jump	All Boys Girls	0.193* 0.256 0.102	0.043 0.217 −0.073	−0.563** −0.727** −0.467**							
Handgrip	All *Boys* *Girls*	−0.037 −0.103 −0.004	−0.002 0.067 −0.053	−0.090 −0.057 −0.114	0.207* 0.113 0.276*						
Flamingo	All Boys Girls	−0.232* −0.375** −0.129	−0.200* −0.301* −0,301*	0.388* 0.504** 0.331*	−0.447** −0.556** −0.405**	−0.084 −0.168 −0.021					
Sit-ups	All Boys Girls	0.213* 0.193 0.208	0.021 0.182 −0.114	−0.420** −0.405** −0.449**	0.475** 0.444** 0.486**	0.101 −0.042 0.250	−0.378** −0.468** −0.325*				
Plate tapping	All Boys Girls	0.000 0.038 −0.051	−0.034 0.008 −0.084	−0.010 −0.138 0.100	0.049 0.275* −0.227	−0.045 0.005 −0.164	0.060 −0.014 0.109	−0.028 −0.003 −0.107			
Sit and reach	All Boys Girls	0.085 0.189 0.078	0.116 0.033 0.157	−0.323** −0.383** −0.344*	0.086 0.229 0.069	0.023 −0.034 0.201	−0.180 −0.055 −0.255	0.074 0.119 0.133	−0.046 0.038 −0.044		
*AP_Math_* *(ρ)*	*All* *Boys* *Girls*	−0.189* −*0.185* −*0.193*	*0.070* *0.177* −*0.066*	−*0.183* −*0.159* −*0.194*	0.247** *0.205* 0.294*	*0.075* −*0.035* *0.205*	−*0.104* −*0.259* *0.099*	*0.169* *0.230* *0.134*	−*0.140* −*0.149* −*0.127*	−*0.014* −*0.083* *0.023*	

Aerobic power calculated from 20 m shuttle run ($\dot{\text{V}}\,{\text{O}}_{2\,p\,e\,a\,k}$) following the Leger protocol (Leger and Lambert, 1982) and expressed in mL⋅kg^–1^ min^–1^; MVPA_OB_, physical activity measured directly using SenseWear Armbands in N = 50 sub-sample (N = 21 boys and 29 girls); AP_math_, academic performance (math grade, corresponding to the final grade awarded at the end of Grade 5); *Significant correlation p < 0.05; **Significant correlation p < 0.01; Italics font represents non-parametric correlations (Spearman rho).

**TABLE 2B:** Correlation coefficients for dependent measures determined for boys and girls in Grade 9.

Grade 9		MVPA_OB_	Shuttle run $\dot{\text{V}}\,{\text{O}}_{2\,p\,e\,a\,k}$	Polygon backward	Standing long jump	Handgrip	Flamingo	Sit-ups	Plate tapping	Sit and reach	AP_Math_
MVPA_OB_	All Boys Girls										
Shuttle run $\dot{\text{V}}\,{\text{O}}_{2\,p\,e\,a\,k}$	All Boys Girls	0.513** 0.407** 0.389**									
Polygon backward	All Boys Girls	−0.283** −0.280** −0.358**	−0.425** −0.686 −0.248								
Standing long jump	All Boys Girls	0.305** 0.167 0.204	0.603** 0.551** 0.546**	−0.321** −0.741** −0.077							
Handgrip	All *Boys* *Girls*	0.251* 0.020 0.221	0.330** 0.211 0.174	−0.137 −0.175 −0.122	0.372** 0.432** 0.154						
Flamingo	All Boys Girls	0.009 −0.137 −0.075	−0.256** −0.432** −0.395**	0.349** 0.435** 0.286*	−0.291** −0.390** −0.442**	0.001 −0.181 −0.010					
Sit-ups	All Boys Girls	0.288** 0.232 0.210	0.576** 0.672** 0.467**	−0.241* −0.469** −0.107	0.588** 0.471** 0.597**	0.325** 0.308* 0.240	−0.229* −0.206 −0.380**				
Plate tapping	All Boys Girls	0.052 0.089 0.014	0.335** 0.369* 0.369**	−0.207* −0.281 −0.144	0.286** 0.372* 0.259	0.224* 0.304* 0.174	−0.094 −0.172 −0.036	0.274** 0.242 0.305*			
Sit and reach	All Boys Girls	−0.168 0.220 −0.129	−0.023 0.231 0.204	−0.206* −0.477** −0.71	0.180 0.408** 0.394**	−0.159 0.034 −0.002	−0.387** −0.485** −0.218	0.149 0.047 0.407**	0.120 0.205 0.102		
*AP_Math_* *(ρ)*	*All* *Boys* *Girls*	0.576** −*0.018* −*0.001*	−*0.128* −*0.056* −*0.182*	−0.541** −*0.249* *0.070*	*0.014* *0.188* −*0.084*	−*0.108* −*0.007* −*0.156*	−*0.041* −*0.213* *0.125*	−*0.188* −*0.149* −*0.189*	−*0.048* −*0.035* −*0.059*	*0.115* *0.214* *0.023*	

Aerobic power calculated from 20 m shuttle run ($\dot{\text{V}}\,{\text{O}}_{2\,p\,e\,a\,k}$) following the Leger protocol (Leger and Lambert, 1982) and expressed in mL⋅kg^–1^ min^–1^; MVPA_OB_, physical activity measured directly using SenseWear Armbands in N = 50 sub-sample (21 boys and 29 girls); AP_math_: academic performance (math grade, corresponding to the final grade awarded at the end of Grade 8); *Significant correlation p < 0.05; **Significant correlation p < 0.01; Italics font represents non-parametric correlations (Spearman rho).

#### Physical Activity and Mathematics Academic Performance

In Grade 6, mathematics grade (AP_math_) was negatively correlated to MVPA_OB_ (*r* = −0.189, *p* = 0.050), such that youth who recorded more PA minutes were also more likely to have lower AP_math_ scores. This relationship was true only when data were combined. Separated by sex, both boys (*r* = 0.185, *p* = 0.176) and girls (*r* = 0.193, *p* = 0.161) demonstrated no relationship between the variables. MVPA_OB_ and AP_math_ were not correlated in Grade 9, either for everyone (*r* = 0.051, *p* = 0.612), for boys (*r* = 0.018, *p* = 0.908) or for girls (*r* = 0.001, *p* = 0.993).

#### Physical Fitness and Mathematics Academic Performance

In Grade 6, mathematics grade AP_math_ demonstrated a low association with standing long jump (*r* = 0.247, *p* = 0.010); however, only in girls were the results of this test significantly correlated (*r* = 0.294, *p* = 0.031). By Grade 9, there were no fitness indicators which correlated significantly to math grade for that year. This was true for both sexes, even for peak aerobic power ($\dot{\text{V}}\,{\text{O}}_{2\,p\,e\,a\,k}$), which did not correlate to AP_math_ in Grade 9.

### Physical Fitness and Activity Predictors of Final Mathematics Grade

A backward stepwise multiple regression was used to determine which indicators may have shared variance to the youths’ final AP_math_ scores, awarded at the end of Grade 9, and based on the delta change scores of the fitness measures. Of all the fitness measures, only backward obstacle course, sit and reach, sit-ups, and delta change in AP_math_ remained significant to the final model [*r*^2^ = 0.494, *F*(4,180) = 43.67, *p* < 0.0001, Tables 3, 4]. When this model was run separately by sex, the input variables differed slightly between boys and girls, wherein shuttle run, handgrip, sit-ups and ΔAP_math_ contributed to the final model for boys [*r*^2^ = 0.495, *F*(4,95) = 23.299, *p* < 0.0001], and only backward obstacle course and ΔAP_math_ contributed to the girls’ model [*r*^2^ = 0.540, *F*(2,81) = 47.7, *p* < 0.0001].

**TABLE 3:** Model summary for the backward stepwise linear regression on the physical fitness change scores from Grade 6 to Grade 9, exclusively.

Model	*R*	*R* ^2^	Adjusted	Std. error of	Sum of	Degrees of	Mean square	*F*-statistic	Significance
			*R* ^2^	the estimate	squares	freedom (df)			
**A**									
1	0.711	0.505	0.408	0.74268	98.020	9	10.891	19.745	0.000
2	0.711	0.505	0.483	0.74057	98.017	8	12.252	22.340	0.000
3	0.711	0.505	0.485	0.73854	97.997	7	14.000	25.666	0.000
4	0.709	0.503	0.486	0.73827	97.522	6	16.254	29.821	0.000
5	0.706	0.498	0.484	0.73948	96.659	5	19.353	35.353	0.000
**6**	**0.703**	**0.494**	**0.483**	**0.74060**	**95.815**	**4**	**23.954**	**43.672**	**0.000**
**B**									
7	0.710	0.504	0.455	0.80156	58.816	9	6.535	10.171	0.000
8	0.710	0.504	0.460	0.79732	58.790	8	7.349	11.560	0.000
9	0.709	0.503	0.465	0.79382	58.667	7	8.381	13.300	0.000
10	0.708	0.501	0.469	0.79097	58.456	6	9.743	15.572	0.000
11	0.707	0.499	0.473	0.78808	58.259	5	11.652	18.761	0.000
**12**	**0.704**	**0.495**	**0.474**	**0.78726**	**57.761**	**4**	**14.440**	**23.299**	**0.000**
**C**									
13	0.751	0.564	0.511	0.67202	43.283	9	4.809	10.649	0.000
14	0.751	0.564	0.518	0.66753	43.282	8	5.410	12.142	0.000
15	0.751	0.564	0.524	0.66316	43.279	7	6.183	14.059	0.000
16	0.751	0.564	0.530	0.65893	43.270	6	7.212	16.610	0.000
17	0.751	0.564	0.536	0.65500	43.238	5	8.648	20.156	0.000
18	0.748	0.559	0.537	0.65423	42.889	4	10.722	25.050	0.000
19	0.745	0.556	0.539	0.65269	42.622	3	14.207	33.350	0.000
**20**	**0.735**	**0.540**	**0.540**	**0.65972**	**41.449**	**2**	**20.724**	**47.617**	**0.000**

*Data were analyzed for (A) entire sample (N = 184), (B) boys (N = 100) and (C) girls (N = 84), respectively. Final models are bolded for interest.*

*Predictors for each model consist of a constant, plus the change scores of: (1): tapping, shuttle run, math score, flamingo, polygon backward, handgrip, sit and reach, sit ups, standing long jump (2) tapping, shuttle run, math score, flamingo, polygon backward, handgrip, sit and reach, sit ups, (3) tapping, shuttle run, math score, polygon backward, handgrip, sit and reach, sit ups (4) tapping, shuttle run, math score, polygon backward, sit and reach, sit ups, (5) shuttle run, math score, polygon backward, sit and reach, sit ups, (6) math score, polygon backward, sit and reach, sit ups, (7) tapping, math score, shuttle run, flamingo, handgrip, polygon backward, sit and reach, sit ups, standing long jump, (8) tapping, math score, shuttle run, flamingo, handgrip, polygon backward, sit and reach, sit ups (9) math score, shuttle run, flamingo, handgrip, polygon backward, sit and reach, sit ups, (10) math score, shuttle run, handgrip, polygon backward, sit and reach, sit ups (11) math score, shuttle run, handgrip, sit and reach, sit ups, (12) math score, shuttle run, handgrip, sit ups, (13) tapping, handgrip, flamingo, shuttle run, polygon backward, math score, sit ups, standing long jump, sit and reach, (14) tapping, handgrip, flamingo, shuttle run, polygon backward, math score, sit ups, sit and reach, (15) tapping, handgrip, shuttle run, polygon backward, math score, sit ups, sit and reach, (16) tapping, shuttle run, polygon backward, math score, sit ups, sit and reach, (17) tapping, polygon backward, math score, sit ups, sit and reach, (18) tapping, polygon backward, math score, sit and reach, (19) tapping, polygon backward, math score, (20) polygon backward, math score. Final model compositions are bolded for interest.*

**TABLE 4:** Coefficients data for the first and final model for (A) entire sample (*N* = 184), (B) boys (*N* = 100), and (C) girls (*N* = 84), respectively.

		Unstandardized coefficients		Standardized coefficients B	*t*-statistic	Significance	95% confidence interval for B		Collinearity Statistics	
Model		*B*	Std. error				Lower bound	Upper bound	Tolerance	VIF
A1	Constant	3.628	0.129		28.070	0.000	3.373	3.883		
	Δ Math grade	0.544	0.043	0.691	12.547	0.000	0.459	0.630	0.953	1.049
	Δ Standing long jump	0.000	0.003	0.005	0.076	0.940	−0.006	0.006	0.609	1.643
	Δ Flamingo	−0.001	0.004	−0.010	−0.193	0.847	−0.008	0.007	0.967	1.034
	Δ Sit ups	−0.018	0.007	−0.163	−2.688	0.008	−0.032	−0.005	0.766	1.305
	Δ Handgrip	0.009	0.011	0.052	0.791	0.430	−0.013	0.031	0.670	1.492
	Δ Polygon backward	−0.031	0.018	−0.096	−1.759	0.080	−0.067	0.004	0.908	1.101
	Δ Sit and reach	0.017	0.010	0.096	1.681	0.095	−0.003	0.036	0.856	1.169
	Δ Shuttle run	0.002	0.002	0.064	1.142	0.244	−0.004	0.016	0.873	1.145
	Δ Tapping	0.006	0.005	0.067	1.170	0.244	−0.004	0.016	0.890	1.124
A6	Constant	3.706	0.115		32.292	0.000	3.480	3.933		
	Δ Math grade	0.546	0.043	0.693	12.790	0.000	0.462	0.630	0.975	1.026
	Δ Sit ups	−0.014	0.006	−0.121	−2.219	0.028	−0.026	−0.002	0.939	1.065
	Δ Polygon backward	−0.029	0.018	−0.090	−1.656	0.099	−0.064	0.006	0.949	1.053
	Δ Sit and reach	0.021	0.009	0.122	2.236	0.027	0.002	0.040	0.930	1.076
B7	Constant	3.526	0.177		19.953	0.000	3.175	3.877		
	Δ Math grade	0.495	0.060	0.641	8.274	0.000	0.376	0.613	0.956	1.046
	Δ Standing long jump	−0.001	0.005	−0.023	−0.199	0.843	−0.011	0.009	0.409	2.446
	Δ Flamingo	−0.002	0.005	−0.037	−0.491	0.624	−0.011	0.007	0.912	1.097
	Δ Sit ups	−0.027	0.010	−0.235	−2.631	0.010	−0.047	−0.007	0.681	1.468
	Δ Handgrip	0.022	0.015	0.141	1.416	0.160	−0.009	0.053	0.561	1.783
	Δ Polygon backward	−0.017	0.027	−0.051	−0.639	0.525	−0.071	0.036	0.824	1.214
	Δ Sit and reach	0.013	0.017	0.069	0.773	0.442	−0.020	0.046	0.702	1.425
	Δ Shuttle run	0.005	0.003	0.161	1.935	0.056	0.000	0.010	0.790	1.265
	Δ Tapping	0.003	0.007	0.040	0.476	0.635	−0.011	0.018	0.751	1.331
B12	Constant	3.638	0.144		25.256	0.000	3.352	3.924		
	Δ Math grade	0.484	0.057	0.628	8.522	0.000	0.372	0.597	0.978	1.022
	Δ Sit ups	−0.025	0.009	−0.216	−2.624	0.010	−0.043	−0.006	0.783	1.276
	Δ Handgrip	0.024	0.012	0.155	1.966	0.052	0.000	0.048	0.857	1.166
	Δ Shuttle run	0.005	0.002	0.155	1.993	0.049	0.000	0.010	0.879	1.137
C13	Constant	3.707	0.215		17.221	0.000	3.278	4.136		
	Δ Math grade	0.605	0.064	0.746	9.409	0.000	0.477	0.733	0.884	1.131
	Δ Standing long jump	0.000	0.005	0.002	0.027	0.978	−0.010	0.102	0.811	1.233
	Δ Flamingo	0.001	0.010	0.006	0.082	0.935	−0.019	0.021	0.896	1.115
	Δ Sit ups	−0.008	0.009	−0.066	−0.821	0.415	−0.026	0.011	0.906	1.103
	Δ Handgrip	−0.003	0.021	−0.011	−0.137	0.891	−0.045	0.039	0.893	1.120
	Δ Polygon backward	−0.057	0.025	−0.177	−2.246	0.028	−0.107	−0.006	0.540	1.852
	Δ Sit and reach	0.014	0.015	0.077	0.915	0.363	−0.016	0.043	0.688	1.453
	Δ Shuttle run	−0.001	0.002	−0.021	−0.2552	0.802	−0.005	0.004	0.849	1.178
	Δ Tapping	0.011	0.007	0.123	1.561	0.123	−0.003	0.026	0.795	1.257
C20	Constant	3.909	0.095		41.187	0.000	3.721	4.098		
	Δ Math grade	0.590	0.061	0.728	9.628	0.000	0.468	0.712	0.930	1.076
	Δ Polygon backward	−0.059	0.024	−0.183	−2.424	0.018	−0.107	−0.010	0.911	1.098

Determining the (possible) effect of MVPA on final AP_math_ scores required a second analysis since MVPA_OB_ were obtained in a sub-sample of youth and not the entire cohort. In this scenario, MVPA_OB_ attained its lowest *p*-value of 0.188, a standardized beta of −0.122, and was thus removed from the model on step 7 of 9 (Tables 5, 6). Likewise, when data were separated by sex, MVPA_OB_ it did not reach significance for either analysis, being removed from the model at step 3 (*p* = 0.698) and step 7 (*p* = 0.315) for boys and girls, respectively, and therefore, only combined data are detailed in subsequent tables.

**TABLE 5:** Model summary for the backward stepwise linear regression on the physical fitness change scores from Grade 6 to Grade 9, with the inclusion of MVPA_OB_.

Model	*R*	*R* ^2^	Adjusted *R*^2^	Std. error of the estimate	Sum of squares	Degrees of freedom (df)	Mean square	*F*-statistic	Significance
1	0.851	0.724	0.625	0.62011	28.207	10	2.821	7.335	0.000
2	0.851	0.724	0.638	0.60954	28.200	9	3.133	8.433	0.000
3	0.849	0.721	0.646	0.60258	28.081	8	3.510	9.667	0.000
4	0.849	0.716	0.652	0.59753	27.906	7	3.978	11.166	0.000
5	0.843	0.711	0.656	0.59379	27.692	6	4.615	13.090	0.000
6	0.837	0.701	0.655	0.59453	27.310	5	5.462	15.453	0.000
**7**	**0.827**	**0.684**	**0.647**	**0.60153**	**26.672**	**4**	**6.668**	**18.428**	0.000
8	0.815	0.664	0.635	0.61196	25.867	3	8.622	23.024	0.000
9	0.799	0.638	0.618	0.61258	24.873	2	12.436	31.749	0.000

*Data were analyzed for the entire sample (N = 39). Model at which point MVPA_OB_ is dropped from the analysis is bolded for interest. Data are presented only for total data set, not stratified by sex.*

*Predictors for each model consist of a constant, plus the change scores of: (1): MVPA, handgrip, shuttle run, standing long jump, polygon backward, sit ups, sit and reach, flamingo, math score, tapping (2) MVPA, shuttle run, standing long jump, polygon backward, sit ups, sit and reach, flamingo, math score, tapping, (3) MVPA, shuttle run, standing long jump, polygon backward, sit ups, sit and reach, flamingo, math score, (4) MVPA, standing long jump, polygon backward, sit ups, sit and reach, flamingo, math score, (5) MVPA, polygon backward, sit ups, sit and reach, flamingo, math score, (6) MVPA, polygon backward, sit ups, flamingo, math score, **(**7) polygon backward, sit ups, flamingo, math score, (8) polygon backward, sit ups, math score, (9) sit ups, math score.*

**TABLE 6:** Coefficients data for the first and final model with the inclusion of MVPA_OB_.

		Unstandardized coefficients		Standardized coefficients *B*	*t*-statistic	Significance	95% confidence interval for B		Collinearity statistics	
Model		*B*	Std. error				Lower bound	Upper bound	Tolerance	VIF
1	Constant	5.029	0.572		8.788	0.000	3.857	6.201		
	Δ Math grade	0.650	0.092	0.786	7.087	0.000	0.462	0.837	0.803	1.245
	Δ Standing long jump	−0.004	0.006	−0.067	−0.644	0.525	−0.016	0.008	0.916	1.092
	Δ Flamingo	0.025	0.017	0.161	1.459	0.156	−0.010	0.059	0.814	1.229
	Δ Sit ups	−0.025	0.014	−0.195	−1.897	0.081	−0.053	0.003	0.850	1.176
	Δ Handgrip	0.004	0.026	0.016	0.140	0.890	−0.049	0.056	0.782	1.279
	Δ Polygon backward	−0.052	0.032	−0.179	−1.640	0.112	−0.117	0.013	0.825	1.213
	Δ Sit and reach	−0.021	0.021	−0.110	−0.997	0.327	−0.065	0.023	0.812	1.231
	Δ Shuttle run	0.003	0.004	0.096	0.819	0.420	−0.004	0.010	0.715	1.399
	Δ Tapping	0.006	0.011	0.066	0.571	0.572	−0.017	0.030	0.746	1.340
9	Constant	4.071	0.137		29.730	0.000	3.793	4.349		
	Δ Math grade	0.621	0.084	0.751	7.415	0.000	0.451	0.791	0.980	1.021
	Δ Sit ups	−0.024	0.013	−0.185	−1.828	0.076	−0.050	0.003	0.980	1.021

*Data were analyzed for the entire sample (N = 39). Data are presented only for total data set, not stratified by sex.*

## Discussion

The results of this study found that longitudinal fitness changes in Slovenian youth were related to changes in objectively assessed physical activity (MVPA_OB_), including small associations between standing long jump and MVPA_OB_. Mathematics score decreased within this 3-year timespan for both boys and girls. The final mathematics grade at the end of primary school was associated with changes in whole-body coordination (backward obstacle course), flexibility (sit and reach) and muscular endurance (sit-ups) when boys’ and girls’ data were combined. When separated, fitness indicators differed such that for boys, aerobic power (shuttle run), upper body strength (handgrip), and muscular endurance (sit-ups) shared variance with final math score whereas whole-body coordination backward obstacle course was the more relevant fitness indicator to girls’ final math score.

### Longitudinal Changes in Physical Activity and Fitness

Physical activity levels in children tend to decrease with age, and a recent meta-analysis has demonstrated that between the ages 11 and 14 (which correspond to grades 6 and 9 in Slovenia), MVPA declines similarly amongst boys and girls at a rate of around 4.3% and 4.5% per year, respectively (Farooq et al., 2020). Analysis from the current investigation, and trends from the ongoing SLOfit surveillance (Jurak et al., 2020), show larger declining trends in Slovenian boys and girls, and greater differences between them. For example, girls experience a ∼15.2% annual decline, thus experiencing 2.6-times larger annual declines in MVPA than boys, who see declines of ∼5.7% annually. Although the declines in MVPA observed between Grades 6 and 9 can be possibly linked to greater school-related workloads (e.g., compulsory annual instruction time increases from 700 to 892.5 h, respectively), and the reduced hours of compulsory physical education after Grade 6 from 105 to 70 h per year, this workload increase and school-related PA is equal for boys and girls, and cannot in and of itself explain the growing sex-effect difference observed in MVPA in the present study.

Similarly, there are growing disparities between boys and girls when observing changes in aerobic power, measured via the shuttle run. In boys, this indicator of cardiorespiratory fitness increased from 45 to 53 mL⋅kg^–1^⋅min^–1^ between Grade 6 and 9, whereas for girls, their values decreased from 46 to 41 mL⋅kg^–1^⋅min^–1^. In the Muscatine study, both boys and girls experienced a decline of VO_2peak_ in this age period (Janz et al., 2000), although in that study, the level of oxygen consumption was much lower than our recorded level, and therefore any changes observed in MVPA for our cohort may have had greater relative effects compared to a less-fit group of children at baseline.

Coherent to the trends in MVPA, boys in our study did not experience a declining trend in any other physical fitness test (other than the flamingo balance test) whilst girls experienced improvement in all physical fitness indicators, despite recorded declines in MVPA. Indeed, girls even exceeded the boys’ performance in terms of the flamingo balance test and sit-and-reach scores, whereas boys achieved higher results in standing long jump and handgrip test. These results suggest that girls may achieve higher levels of neuromuscular fitness by age 14 compared to their male counterparts, which could be linked to their earlier timing of puberty/maturation, and consequently more developed central nervous system at this age (Hoyt et al., 2020). Unfortunately, direct assessment of maturation was outside of the scope of this study, so any improvements in these measures must be considered when interpreting the data. In flexibility, girls also tended to achieve higher results throughout childhood and adolescence. By age 14, boys tended to achieve higher levels of muscular fitness which is linked to increasing muscle mass and consequent muscle power, likely because of higher testosterone levels as puberty continues to progress (Handelsman, 2017).

### Mathematics Academic Performance in Primary School

In the present study, mathematics grades decreased between Grade 6 and Grade 9 from ∼4.2 to ∼3.5. This magnitude of decrease is not surprising, given that Grade 5 is the last year a generalized education teacher is the one who administers the mathematics curricula. By Grade 6, the youth are taught by a specialist mathematics teacher, and both the volume of work and difficulty level are increased dramatically compared to early primary schooling periods. Population data from national assessments indicate that a typical decrease in math grade does occur, with the average score at the end of Grade 9 being 3.3 (Perger, 2021). Therefore, our results are in-line with national standards for this academic performance metric.

### Physical Fitness and Academic Performance in Children and Youth

Of all the fitness indicators, positive AP is often specifically associated with higher aerobic function (Sardinha et al., 2016). For example, on a sample of boys from Spain (Torrijos-Niño et al., 2014), the authors found that AP was positively related to aerobic fitness, and that obese boys had lower AP scores compared to overweight or normal weight boys. Unsurprisingly, their data suggest that ‘academically superior’ PF in children and youth are likely the result of lifestyle patterns, including discipline in school-related work and habitual daily PA, a pattern of evidence also reflected in other studies (Donnelly et al., 2016). Currently, although shuttle run (and by extension, aerobic power) epidemiological literature for children and youth is very well-documented (Tomkinson et al., 2017; Lang et al., 2019), there are few examples where negative fitness and obesity trends have been reversed, especially over the past 20 years. To this point, the Republic of Slovenia has demonstrated a net increase in CRF between 1993 and 2013 in both boys and girls, despite initial decreases observed from 1993 to 2003 (Morrison et al., 2021). Indeed, Slovenian schoolchildren universally meet or exceed international standards for CRF cut-off values for minimizing future cardiovascular health risk. Whether this higher universal fitness standard affects AP in these children and youth are yet to be determined, but the present study did not relate shuttle run aerobic power to mathematics grade *per se* for either sex within a given grade. Instead, the 3-year longitudinal changes observed in aerobic power were a predictor for final math score for boys (but not for girls), indicating both a possible reduction in statistical power when the regression was split by sex, and also the interaction effect present in that metric, where boys saw a net increase in relative function between Grade 6 to Grade 9 (from 46 ± 26 to 64 ± 25 mL^–1^⋅kg^–1^⋅min^–1^), whereas girls demonstrated the reverse trend (from 46 ± 27 to 42 ± 20 mL^–1^⋅kg^–1^⋅min^–1^). It would be interesting to compare these longitudinal changes in PF and AP in Slovenian schoolchildren to others across Europe, the OECD, and internationally, to determine to what degree increasing PF directly improves AP in a dose-response manner, especially for children who may be starting from a more sedentary and lower aerobic fitness standard.

Previous meta-analyses have found that, in addition to cardiorespiratory fitness, speed-agility, motor coordination, and perceptual motor skills are each highly associated with AP (Ruiz-Ariza et al., 2017), but evidence for strength and flexibility remained unclear. Certainly, some authors consider that motor competence is ‘indissoluble’ from cognitive competence, a model in which cognitive skills arise from motor action (AVILÉS et al., 2014), with motor coordination being one of the most important factors to the evolutionary development of children (Fernandes et al., 2016; Ruiz-Pérez et al., 2016). The present study found a small proportion of the variance in final math grade could be attributed to physical fitness components encompassing motor coordination (backward obstacle course), flexibility (sit-and-reach) and strength (sit-ups). These results support the theory of association between body-kinaesthetic intelligence and coordinative performance, which is further reinforced by neural connections existing across structural/muscular factors (Diamond and Lee, 2011). Sit-ups may have contributed to the final model because it is an isokinetic endurance test which considers not only a child’s physical abilities, but also psychological characteristics like persistence, a necessary component to any learning process. Our results are consistent with a similar analysis conducted on a sample of boys from Spain (Torrijos-Niño et al., 2014), and a study where the odds ratio for effects of PF improvement on AP, and mathematics specifically, were calculated for highly fit and unfit children (Sardinha et al., 2016).

In our analysis, we were not able to prove the interrelatedness of objectively assessed PA on AP, which proposes that PF indicators may be more insightful than PA alone when studying this phenomenon. Firstly, the information on MVPA alone cannot indicate whether a person is fit or not. This is related to the second limitation of reliability of MVPA assessment, namely, that despite the established recommendations that MVPA should be recorded for several consecutive days, this does not guarantee that actual PA is representative for long-term habitual PA during the observed days. In this regard, PF could serve as a more reliable indicator of habitual PA, since the level of PF is the direct result of long-term habitual PA as an important determinant of phenotype (Sallis et al., 1993a,b). Indeed, due to interpersonal differences in body metabolism, individuals require different frequency, intensity, and duration of PA to obtain or preserve certain levels of PF, or related health benefits (Oja, 2001). Thirdly, PF seems to be more direct indicator of physiological functioning of human organism which determines also cognitive functioning than PA, and also the effect of PA is itself moderated by PF (Lopes and Rodrigues, 2021).

### Espousing Systematic Physical Fitness Monitoring in the Young

There is clear evidence that monitoring PF in children can be critical to maintaining this key health indicator (Ortega et al., 2008). Higher levels of PF are associated with greater PA (Wang, 2019), including better socialization (Li and Li, 2018) and academic performance (Sember et al., 2020b) and indeed, fitness testing is much more than just ‘one more school assessment’ since it helps increase awareness of how one’s body moves through space. How fit a child is now can relate to how fit and active they become as adults (Kvaavik et al., 2009). Those who have high ‘physical literacy’ (i.e., are attuned to how their body works and what it needs to function properly), are better able to foster life-long physical activity habits (Whitehead, 2010). Importantly, skill-based fitness developments are legitimate manifestations of physical literacy development since physical literacy is a multidimensional and interactive construct comprising of the physical, behavior, cognitive and affective domain (Whitehead, 2010). Thus, PF is a critical component to physical literacy overall. Additionally, monitoring fitness in schools is an effective policy making tool since there are well-educated professionals (teachers and others) who can ensure effective and accurate student fitness measurements in a timely and accurate fashion (e.g., SLOfit) (Jurak et al., 2019). Monitoring fitness as children mature offers education and public health decision-makers opportunity to respond quickly and effectively to changes in child PF trends, a critical pillar of any public health strategy since globally, children have become more inactive and overweight (Aubert et al., 2018).

It is important to note that fitness testing *per se* should never be used to grade the student. The assessment of PF is conducted to provide professionals with accurate measures of educational achievement, the health and functional status of the children, and can act as an operational starting point for setting individual goals and tailoring curricula to individual needs. It must be clearly communicated as such to the children as well so that they treat these examinations in context. Timely testing allows for professionals to gain an understanding of the child’s educational development and health status to make informed decisions regarding education or further treatment (Lloyd et al., 2010). This is an appropriate way to run effective physical education classes, where students understand the rationale for standardized testing.

Data from the current investigation were possible only because there is a concerted, ongoing effort to chart the fitness changes of the Slovenian schoolchildren population to inform policy making decisions and affect positive public health decisions. Data from fitness databases in Slovenia have contributed to finding that there was a reversal in the negative trends of child aerobic fitness (Morrison et al., 2021), charting pediatric rates of obesity (Potoènik et al., 2020), and identifying how the COVID-19 pandemic has affected child fitness overall (Jurak et al., 2021). By utilizing near real-time health status changes in youth, this study demonstrates how implementing a robust fitness surveillance monitoring program at a population level can detect meaningful changes in health status, even when societal and global perturbations dramatically alter civic life (e.g., conflicts, pandemics, and recessions). Often during times of social transition, decreases in opportunities to engage in quality physical activity may diminish one’s physical fitness, especially for vulnerable populations, like the young. Data from the current investigation underscore that a variety of physical fitness indicators are related to changes in academic performance, especially for mathematics grade, and these fitness markers are heterogeneous between boys and girls. Only by continuously monitoring fitness can one make changes to regional, national, and international policies which can then have lasting trickle-down effects on longstanding societal health issues like obesity, chronic hypertension, stroke, as well as effects like cognition, academic performance, fitness, and mental health.

## Conclusion

Girls and boys in Slovenia are becoming less physically active when progressing from Grade 6 to Grade 9. Decreases are also evident for AP in mathematics in both girls and boys during this timeframe, likely reflecting structural issues present within the Slovenian primary education system (i.e., students aged ∼6–14 years), rather than other external or physical fitness factors alone. In contrast to prevailing existing evidence which link AP with cardiorespiratory fitness lower math grades in our study are also moderately associated with indicators of muscular and neuro-muscular fitness, including changes in whole-body coordination, flexibility, and muscular endurance for the entire sample. Aerobic power, upper body strength, and muscular endurance share more common variance to final math grade in boys, whereas whole-body coordination was the more relevant index in girls; this finding suggests that future research exploring the relationship of AP and PF should not be limited to cardiorespiratory PF and should also encompass muscular and neuro-muscular components of PF. Our study suggests that PF can serve as more useful indicator for assessment of AP risks than PA, but also that the current data indicates the association between PF and PA declines with age. The reason of this decline remains unclear but directs researchers that toward adolescence, factors other than PF may be more detrimental to AP in general, and mathematics grade specifically.

## Data Availability Statement

The raw data supporting the conclusions of this article will be made available by the authors, without undue reservation, to any qualified researcher.

## Ethics Statement

The studies involving human participants were reviewed and approved by the Slovenian National Medical Ethics Committee (ID:138/05/13), following the Declaration of Helsinki. Written informed consent was obtained from the minor’s legal guardian/next of kin (and assent from the child) to participate in the study.

## Author Contributions

VS: investigation, conceptualization, formal analysis, visualization, writing—original draft, writing—review and editing, and writing—approval of final manuscript. GJ: project administration, investigation, conceptualization, resources, writing—review and editing, and writing—approval of final manuscript. GS: project administration, investigation, conceptualization, resources, writing—review and editing, and writing—approval of final manuscript. SM: conceptualization, formal analysis, visualization, writing—original draft, writing—review and editing, and writing—approval of final manuscript. All authors contributed to the article and approved the submitted version.

## Conflict of Interest

The authors declare that the research was conducted in the absence of any commercial or financial relationships that could be construed as a potential conflict of interest. The handling editor declared a past co-authorship with one of the author VS.

## Publisher’s Note

All claims expressed in this article are solely those of the authors and do not necessarily represent those of their affiliated organizations, or those of the publisher, the editors and the reviewers. Any product that may be evaluated in this article, or claim that may be made by its manufacturer, is not guaranteed or endorsed by the publisher.
